# Team based collaborative care model, facilitated by a mHealth enabled and trained nurse, for management of heart failure in India (TIME-HF): design and rationale of a parallel group, open label, multi-centric cluster randomized controlled trial

**DOI:** 10.12688/wellcomeopenres.19196.1

**Published:** 2023-05-03

**Authors:** Panniyammakal Jeemon, Charantharalyil Gopalan Bahuleyan, Devaraju Chandgalu Javaregowda, Eapen Punnoose, Gopalan Rajendiran, Govindan Unni, Jabir Abdullakutty, Jayakumar Balakrishnan, Johny Joseph, Justin Paul Gnanaraj, Madhu Sreedharan, Meera R Pillai, Neenumol KR, Paul Thomas, Placid Sebastian, Rachel Daniel, Rajeev Edakutty, Sajan Ahmad, Shafeeq Mattummal, Sunu C Thomas, Stigi Joseph, Sunil Pisharody, Susanna Chacko, N Syam, Tiny Nair, Veena Nanjappa, Vijayan Ganesan, Vijo George, Sanjay Ganapathi, Sivadasanpillai Harikrishnan

**Affiliations:** 1 Sree Chitra Tirunal Institute for Medical Sciences and Technology, Thiruvananthapuram, Kerala, 695011, India; 2 Ananthapuri Hospitals and Research Institute, Thiruvananthapuram, Kerala, India; 3 Sri Jayadeva Institute of Cardiovascular Science and Research, Mysore, Karnataka, India; 4 Malankara Orthodox Syrian Church Medical College, Kolenchery, Kerala, India; 5 PSG Institute of Medical Sciences, Coimbatore, Tamilnadu, India; 6 Jubilee Mission medical College and Research Institute, Thrissur, Kerala, India; 7 Lisie Hospital, Ernakulam, Kerala, India; 8 Thangam Hospital of PMRC, Palakkad, Kerala, India; 9 Caritas Hospital, Kottayam, Kerala, India; 10 Rajiv Gandhi Government General Hospital, Madras Medical College, Chennai, Tamilnadu, India; 11 NIMS Heart Foundation, NIMS Medicity, Thiruvananthapuram, Kerala, India; 12 KIMS Hospital, Thiruvananthapuram, Kerala, India; 13 General Hospital, Ernakulam, Kerala, India; 14 Aster MIMS, Kannur, Kerala, India; 15 NS Memorial Institute of Medical Sciences, Kollam, Kerala, India; 16 District Cooperative Hospital, Calicut, Kerala, India; 17 St Gregorios Memorial Mission Hospital, Parumala, Kerala, India; 18 Aster MIMS, Calicut, Kerala, India; 19 Little Flower Hospital and Research Centre, Angamaly, Ernakulam, Kerala, India; 20 EMS Memorial Cooperative Hospital and Research Centre Ltd, Malappuram, Kerala, India; 21 Government District Hospital, Kollam, Kerala, India; 22 PRS Hospital, Thiruvananthapuram, Kerala, India

**Keywords:** Heart failure, collaborative care model, mhealth application, cluster randomised controlled trial

## Abstract

**Background: **Heart failure (HF) is a debilitating condition associated with enormous public health burden. Management of HF is complex as it requires care and coordination with different cadres of health care providers. In resource poor settings, physician driven initiatives to improve HF management is difficult due to multiple barriers. We propose to develop a team based collaborative care model (CCM), facilitated by a trained nurse, for management of HF with the support of mHealth and evaluate their acceptability and effectiveness in Indian setting.

**Methods: **The proposed study will use mixed-methods research for assessment. A formative qualitative research will identify barriers and facilitators for implementing CCM for the management of HF and develop components of the intervention. Subsequently, a cluster randomised controlled trial (RCT) involving 22 centres (tertiary hospitals) with more than 1500 HF patients will be conducted to assess the effectiveness of the CCM in improving the overall survival as well as days alive and out of hospital (DAOH) at two-years (CTRI/2021/11/037797). The DAOH will be calculated by subtracting days in hospital and days from death until end of study follow-up from the total follow-up time. Poisson regression with a robust variance estimate and an offset term to account for clustering will be employed in the analyses of DAOH. A rate ratio and its 95% confidence interval (CI) will be estimated. The scalability of the proposed intervention model will be assessed through economic analyses (cost-effectiveness) and the acceptability of the intervention at both the provider and patient level will be understood through process evaluation.

**Potential Impact: **The TIME-HF trial will provide evidence on whether a nurse-led team based CCM with mHealth support is effective in improving the clinical outcomes of HF in India. The findings may change the practice of management of HF in financially constrained low and middle-income countries.

## Introduction

Heart failure (HF) is a major public health problem with increased morbidity and mortality. The number of individuals with HF accounts for 1.3 to 4.6 million in 2010, in India ^1– 3^. Additionally, the number of deaths due to HF in India from 1990 to 2013 showed an increase of 138 percent ^2^. The best available estimates suggest that the incidence of HF in India is 1 per 1000 population ^2^. The predominant etiology of HF in India is ischemic heart disease (IHD) ^4^. Due to the steady increase in the absolute number of individuals with IHD over the last three decades ^5, 6^, HF burden is expected to grow substantially in India.

Heart failure patients in India receive sub-optimal treatment and experience high mortality. For example, only one of four eligible HF patients received guideline-directed medical therapy (GDMT) at discharge ^2, 7^. Similarly, one of three patients adhered to GDMT in a large study in India involving 15,870 patients with reduced left ventricular ejection fraction (EF<40%) ^8^. Additionally, one of five patients died within three months of follow-up ^2^. The long-term prognosis of HF is also poor with a median survival time of 3.7 years ^9^. However, those who received GDMT experienced lower mortality and survived longer ^2^. Data from the Kerala Heart Failure Registry ^10^, and the National Heart Failure Registry ^4^ clearly demonstrate survival benefits in patients who received GDMT during index hospitalisation. Further, the survival benefits of GDMT persisted up to five years of follow-up ^9^.

Physician driven quality improvement initiatives in HF management may not be feasible, scalable, and effective in India. The PINNACLE India quality improvement programme concluded that in a country with a disproportionate provider/patient ratio and low levels of government funding for quality improvement, physician-driven initiatives for practice-based learning and improvement are extremely difficult ^11^. The key barriers include lack of electronic medical records, virtually non-existent outpatient record-keeping, and difficulty of engaging physicians due to their busy clinical schedules ^11^. To overcome these barriers, we propose a task sharing strategy of empowering trained nurses as facilitators of HF care in India. 

A specially trained nurse facilitating the management of HF is a viable alternative strategy ^12– 15^. In general, a specialist HF nurse shares the role of a physician, assess the patients, and manage them based on the tested protocols/algorithms. Additional role of nurses includes psycho-social aspects of self-management of the condition in home settings ^16^, communicating self-care guidelines ^15^, and regular monitoring of patient conditions even when they are away from the hospital or outpatient settings. These strategies in general were effective in achieving improved physical functioning, reduced hospital length of stay and increased adherence towards pharmacological therapy in high-income settings. Three recent meta-analyses also show that task sharing strategy, especially involving nurses in management of cardiovascular risk conditions such as hypertension ^17^, dyslipidemia ^18^ and diabetes is effective in achieving desirable outcomes in low and middle-income countries (LMIC) as well.

The collaborative care model (CCM) based on Wagner’s Chronic Care Model ^19^ is proposed as a key strategy in the management of HF. For example, CCM resulted in improvements in hospitalisation rates ^20, 21^ and quality of life ^22^, and reduction in cost associated with management of HF ^23– 26^. Additionally, increased use of GDMT and improved self-care are also attributed to interventions based on CCM ^27, 28^. However, most of them are small single centre studies leading to reduced reliability of their findings. We propose to develop a team based CCM, facilitated by a trained nurse, for management of HF and evaluate its acceptability and effectiveness in the Indian settings.

The major aims of the study are as follows: (1) to identify barriers and facilitators for implementing a team based CCM for the management of heart failure, (2) to assess the effectiveness of the CCM in improving the days alive and out of hospital (DAOH) at two-year follow up in patients with heart failure. The DAOH is a patient-centred outcome, which accounts for multiple events over the two-year course of a study period, weighs death more than hospitalization, and deaths occurring early more than those occurring later. The multi-component quality improvement intervention will utilise a team-based CCM including risk-based management, mHealth for real time monitoring of warning signs/symptoms and patient/family caregiver involvement. and (3) to evaluate the scalability of proposed intervention model (a) the overall *cost-effectiveness* of the intervention strategy, (b) *at the provider level*, the ease of using the protocols/mHealth application, impact on work load, and satisfaction, and (c) *at the patient level*, risk-perception, ease of seeking health care, utility of understanding risk and attending to warning signs/symptoms, changes made to health behaviours and adherence to guideline directed therapies.

## Methods

### Ethical Considerations

The participants will be informed about the study and provided with a detailed information sheet ^29^. Trained research nurse appointed by the principal investigator will obtain written informed consent from all study participants. The research study is approved by institutional ethics committee of Sree Chitra Tirunal Institute for Medical Sciences and Technology (SCTIMST) and of the participating centres (SCT/IEC/1691/AUGUST-2021). The study protocol is registered with the clinical trial registry clinicaltrials.gov (CTRI/2021/11/037797). All changes in the trial protocol will be informed to the institutional review boards.

All serious adverse events will be reported immediately to the respective ethics committee of the participating centres and the study co-ordinating centre (SCTIMST). The principal investigators (PIs) and Co-PIs will have the access to the final data set. Public access to the data set after de-identification will be provided upon formal request with necessary permission from the SCTIMST ethics committee after three years from the date of completion of the study.

### Study design approach

The proposed study will use mixed methods to achieve the study aims. The design approaches will include: Aim 1- formative qualitative research, Aim 2- a multi-centric cluster randomised controlled trial, and Aim 3- cost effectiveness and evaluative qualitative research. We will follow the Medical Research Council (UK) guidance on developing and evaluating complex interventions ^30^ and guidance for reporting intervention development studies in health research ^31^. The trial duration will be from September 2021 to August 2026 ^32^.

The protocol design is based on the Standard Protocol Items: Recommendations for Intervention Trials (SPIRIT) Checklist ^33^. The SPIRIT flow chart shows the schedule of enrolment, interventions, and assessments of TIME-HF trial ^34^.

***Formative qualitative research (Aim 1)***

In-depth interviews with multiple stakeholders like patients, carers, nurses, community health workers, primary care physicians, and cardiologists will be conducted ( Table 1). Interviews with nurses and cardiologists will be face-to-face, semi-structured and include questions regarding HF care integration. Additional in-depth interviews will be conducted with dieticians, specialist physiotherapist and clinical psychologists to get their perceptions of CCM. In-depth interviews with adult HF patients will gather information on their understanding of diseases, barriers and facilitators to care, and feedback on the proposed intervention components (lifestyle education, disease management program, pharmacologic treatment, self-care, and care coordination). Similarly, in-depth interviews of adult carers of patients with HF will gather information on self-management and care coordination. The intervention and trial protocols will be modified to incorporate key findings collected from qualitative interviews to make the intervention more contextually relevant.

**Table 1.** Formative qualitative methods.

Participants	Methods/Number	Topics
Patients with HF both male and female in various age groups	In-depth interview Total 10-12 IDIs	● Perceptions and behaviours on clinical management, lifestyle, and self-care ● Feedback on proposed intervention components and methods ● Assess patients’ interest and acceptability
Caregivers of patients with HF	In-depth interview Total 10-12 IDIs	● Perceptions and behaviours on clinical management, lifestyle, and self-care ● Feedback on proposed intervention components and methods ● Gauge caregivers’ interest and acceptability
Physicians Nurses Dieticians Physiotherapists	In-depth interview 10-12 interviews in each category (30-36 total interviews)	● Perceived quality of HF care ● Perceived patient barriers and facilitators to delivery of care, lifestyle change, and self-care ● Gauge feasibility of planned intervention components

***Collaborative care model intervention (Aim 2)***

A parallel group cluster RCT of more than 1500 adult HF patients from 22 units in India ^35^ will be used to address Aim 2. Each participating unit will be randomly assigned to one of two arms: 1) those delivering a comprehensive care model (CCM) (intervention) or 2) those delivering standard of care (usual care).

Cluster eligibility criteria: Eligible units will include HF centres of major hospitals from the national heart failure registry or Kerala heart failure registry that serve ≥80 new patients every six-months and consent to the study randomisation plan. The study units will be selected based on their willingness to participate in this study. All HF patients will also be required to provide individual consent to participate. The selected sites will be requested to recruit consecutive patients during the study recruitment phase.

Patient eligibility criteria and randomisation: Potential participants must meet the standard definition of HF ^36^ (HF with reduced ejection fraction of <40%) based on echocardiography to be eligible to enter the study. Consecutive patients will be recruited from the participating centres. Eligible and consenting units will be randomized at one time point, prior to trial implementation. The allocation ratio will be 1:1. Randomisation procedures will not be blocked, restricted, or matched. We will use computer generated random numbers to allocate half of the units to each arm.

*Duration of treatment period and follow-up*

We will conduct a rolling recruitment over a period of six-nine months. Each patient in the study will be followed-up for a period of two years from the date of recruitment. The first follow-up visit in the intervention arm will be conducted on the seventh day. Additional two follow-up visits for clinical status occurs at an interval of three-months. Participants are subsequently seen no less than every six months. Regardless of the treatment group assigned, we will follow-up all study participants in this manner (assessment at 7-th day will be only in the intervention arm) until study completion.

*Measurements in the study*

A structured questionnaire ^37, 38^ will be administered by trained research nurse to collect relevant data at baseline, and every three months until two years from baseline. At these visits, interval assessments of HF and angina symptom status, current use of medications, and clinical endpoint data including hospitalisations and procedures since the previous visit will be documented ( Table 2). The baseline questionnaire includes assessments of demographic and socio-economic variables, general health status, aetiology, history and risk factors, diet pattern, physical activity, tobacco, and alcohol consumption quality of life, six minutes walking distance. In follow-up visits depression and anxiety scores will be collected from each participant by using Patient Health Questionnaire- 9 (PHQ-9) ^39^ and Generalized Anxiety Disorder Questionnaire (GAD-7) ^40^. Medication adherence will be measured using four item Morisky Green Levine medication adherence scale ^41^. The study questionnaire will also assess the patient satisfaction in quality of care, family support and patient care in depression.

**Table 2.** Study measurements in TIME-HF.

Study measurements	Method/Instrument
Blood pressure in mmHg	Electronic BP monitor
Height in cm	Stadiometer
Weight in kg	Digital weighing scale
Waist circumference in inches	Non elastic measuring tapes
Etiology and risk factors	Details of the etiology and risk factors using a questionnaire
Investigations	Blood reports, ECHO, ECG reports
Current Medication	Details of all medications the patient was taking at the time of contact
Depression	PHQ-9 ^39^
Anxiety	GAD-7 ^40^
Self-management Medication adherence, weight, family support, diet, depression care	Morisky, Green and Levine medication adherence scale ^41^, weight management, Diet Management, Family support; Patient care in depression
Quality of life	KCCQ ^42^, EQ-5D-5L ^43, 45^, 6 Minute Walk Test ^44^
Walking ability	6 Minute Walk Test ^44^
Physical disability (muscular strength)	Hand grip strength- Dynamometer
Frailty	Fried frailty index ^46, 47^
Functional capacity	Specific Activity Questionnaire
Physical activity intensity	The Borg Scale of Perceived Exertion ^48^
Disease severity	Modified Borg Dyspnea Scale during 6MWT (0 - 10) ^49, 50^, Responsiveness to change in heart failure symptoms
Treatment burden	MTBQ ^51^
Patient satisfaction	Patient satisfaction in Quality of care, Care transitions measure (CTM3) ^caretransitions.org/^, B-Prepared Scale ^52^

Kansas City Cardiomyopathy Questionnaire (KCCQ) ^42^ and EQ-5D-5L ^43^ will be used to measure quality of life. KCCQ is a disease specific tool to measure quality of life. The EQ-5D-5L is a standardised validated instrument for assessing the functional status, severity of disease and treatment.

A standard adjustable handle constant handgrip dynamometer will be used to measure handgrip strength. Three measurements will be taken in dominant and non-dominant hand. The average of the highest value of dominant and non-dominant handgrip strength will be used in the analysis.

The six-minute walk test (6MWT) will be measured during the baseline and follow-up visits by following the standard protocol ^44^. Before 6MWT the research nurse will measure the blood pressure, pulse, and oxygen level usually with a pulse oximeter. During the test the individual will have to walk for six minutes. After the test, staff will record the distance covered along with oxygen level, pulse rate and post walk Borg dyspnoea levels.

Multimorbidity treatment burden will be assessed by using multimorbidity treatment burden questionnaire (MTBQ) ^51^. Health care expenditure data will be collected every six months. Clinic based blood pressure will be obtained during clinic visits. Blood will be collected six monthly to assess renal function and serum electrolytes. Further, we will measure beta-natriuretic peptides at baseline, six-month interval follow-up visits if they are routinely done as part of the patient care. The detailed study measurements at baseline and during the study period are explained in Table 3.

**Table 3.** Measurement of different parameters at baseline (B), and during follow-up at 3 months (F 3M), 6 months (F 6M), 9 months(F 9M), 12 months(F 12M), 15 months(F 15M), 18 months(F 18M), 21 months(F 21M), 24 months(F 24M).

Study Measurements	B	F 3M	F 6M	F 9M	F 12M	F 15M	F 18M	F 21M	F 24M
Blood Pressure (in mmHg)	✔	✔	✔	✔	✔	✔	✔	✔	✔
Height (in cm)	✔	✔	✔	✔	✔	✔	✔	✔	✔
Weight (in kg)	✔	✔	✔	✔	✔	✔	✔	✔	✔
Waist circumference (in inches)	✔	✔	✔	✔	✔	✔	✔	✔	✔
PHQ-9	✔								
Patient care in depression	✔	✔	✔	✔	✔	✔	✔	✔	✔
KCCQ 12	✔								
GAD-7	✔								
Morisky, Green and Levine medication adherence scale		✔	✔	✔	✔	✔	✔	✔	✔
MTBQ		✔	✔	✔	✔	✔	✔	✔	✔
Diet Management		✔	✔	✔	✔	✔	✔	✔	✔
Fried frailty index	✔	✔	✔	✔	✔	✔	✔	✔	✔
6MWT	✔	✔	✔	✔	✔	✔	✔	✔	✔
Modified Borg dyspnoea scale	✔	✔	✔	✔	✔	✔	✔	✔	✔
Hand grip strength	✔	✔	✔	✔	✔	✔	✔	✔	✔
Specific activity questionnaire		✔	✔	✔	✔	✔	✔	✔	✔
Borg scale of perceived exertion		✔	✔	✔	✔	✔	✔	✔	✔
weight management		✔	✔	✔	✔	✔	✔	✔	✔
EQ-5D-5L	✔	✔	✔	✔	✔	✔	✔	✔	✔
Responsiveness to change		✔	✔	✔	✔	✔	✔	✔	✔
Family support		✔	✔	✔	✔	✔	✔	✔	✔
Responsiveness assessment		✔	✔	✔	✔	✔	✔	✔	✔
Patient satisfaction in quality of care	✔	✔	✔	✔	✔	✔	✔	✔	✔
CTM-3	✔								
B-prepared scale	✔								
Treatment expenditure questionnaire			✔		✔		✔		✔

*Study intervention overview*

All patients receiving care at a unit randomised to the enhanced usual care arm will receive the same standard of care, while all patients receiving care at units randomised to the intervention arm will receive the CCM based care.

Usual care arm: The treating physician will provide care for patients at the units that are randomised to the usual care arm. However, the physician will be assisted by a clinical coordinator to capture relevant demographic, clinical and biochemical variables of interest, and data regarding cost of care, patient satisfaction and quality of life by using a structured interview schedule.

**Figure 1.**
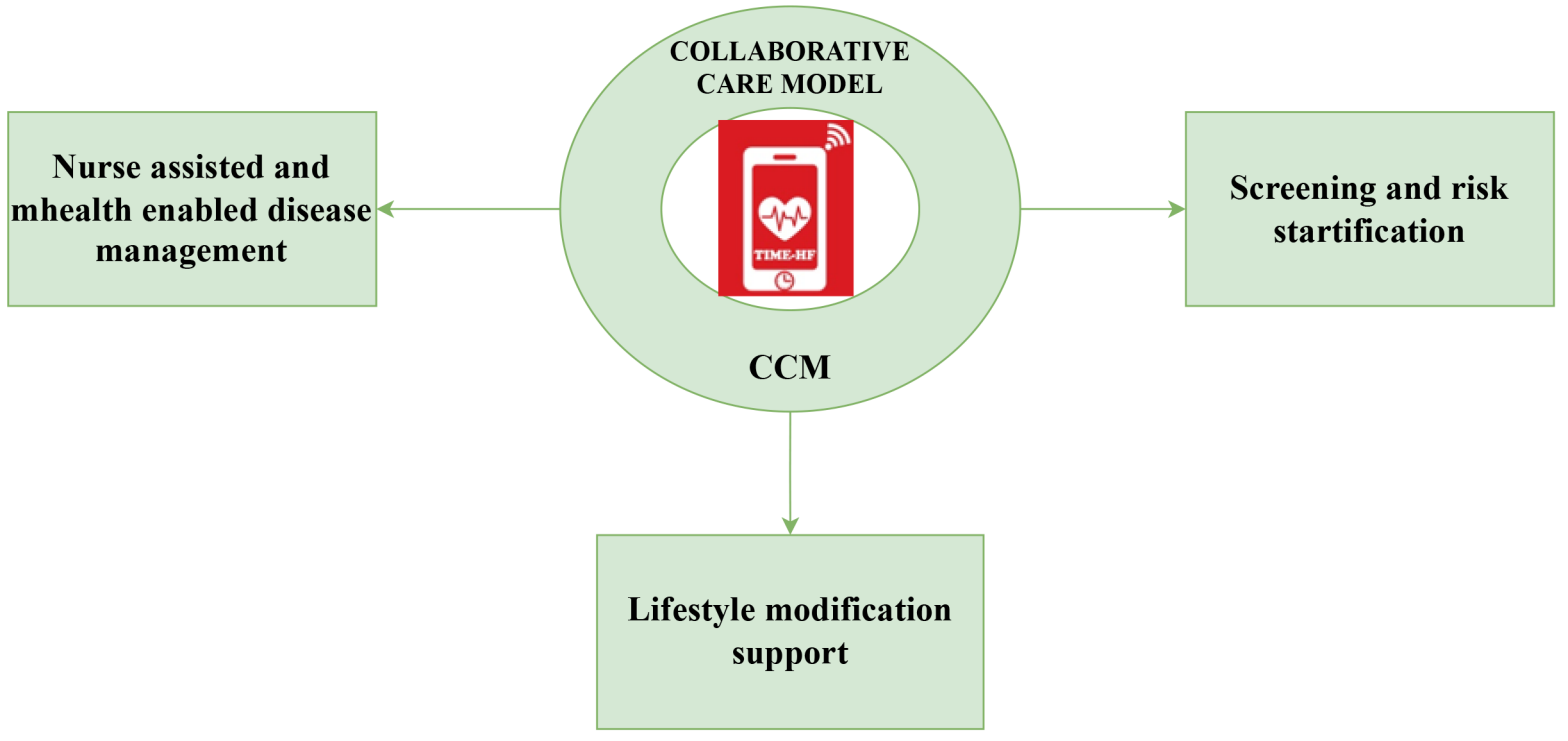
Collaborative care model (CCM) interventions in TIME-HF trial.

**Figure 2.**
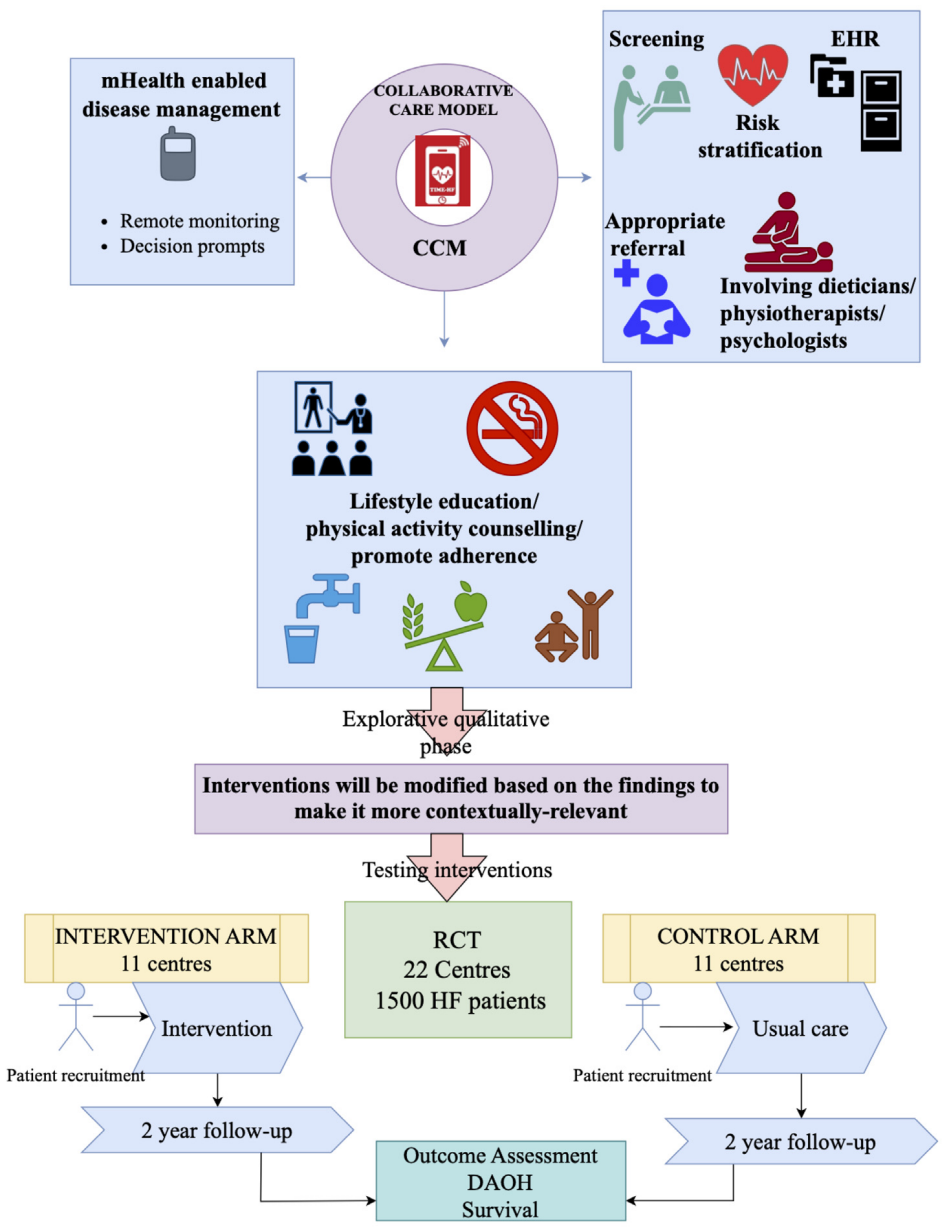
Overview of the study intervention in TIME-HF.

Intervention arm: The intervention will leverage the existing management practices at each site to deliver a comprehensive, integrated HF care led by a trained nurse with support from physicians, dieticians, physiotherapist, and clinical psychologists ( Figure 1). Information from the formative research will be scientifically integrated into the comprehensive management programme. The nurses will be enabled with mHealth technology to facilitate the care delivery. The intervention will consists of three phases ( Figure 2); 1) screening and risk stratification, 2) lifestyle modification support (nutritional education, tobacco and alcohol cessation, exercise or activity planning, daily weight monitoring, assessment of the need for cognitive behavioural therapy, and identification of warning clinical signs) and pharmacologic management, and 3) nurse facilitated and mHealth assisted disease management program, self-care management program, active follow-up and continuous monitoring of clinical conditions of the patient while the patient is away from the hospital settings.

***Evaluate the scalability of proposed intervention model (Aim 3)***

*Cost effectiveness and acceptability analyses*

Information for economic analyses will be collected during the trial to obtain detailed resource consumption. A top-down approach will capture implementation costs from a health systems perspective by quantifying the resources deployed. Some of these will be once only activities (e.g., development of a mobile telephone application) but most will be recurring (e.g., nurse hours per year). We will then cost these resources according to unit costs in India. A bottom-up approach will collect patient level out of pocket expenditure and other personal financial losses or gains. It will include human resource costs, doctor visits, laboratory tests, cost of medications, and co-payments for medications. Rates of outpatient (specialty clinic) visits, hospitalisations, and clinical events will also be obtained. Indirect costs (travel, missed work time, and lost productivity) incurred by patients will be estimated using questionnaires that have been used in previous South Asian studies. It will be estimated using questionnaires administered at every six months study visits.

At the end of treatment, patients and nurses will be asked about quality of care, intervention barriers, side effects, and perceived participation benefits/disadvantages ( Table 4). Additionally, by using a close-out questionnaire, we will also ask patients to self-report their compliance to individual elements of the intervention. Data from follow-up visits questionnaire will also capture key quality indicators ( Table 5). The key domains of quality will include access, timeliness, safety, patient centredness, and equity. We will describe fidelity to the intervention package components, types of changes made by healthcare organisations, how the changes were implemented, and identify multi-level contextual factors and causal pathways that affect implementation, process, and outcomes. We will use triangulation to integrate close-out questionnaire, follow-up questionnaire and qualitative process outcomes data.

**Table 4.** Qualitative methods to understand the reach and acceptability of interventions.

Participants	Methods/Number	Topics
Patients with HF both male and female in various age groups	In-depth interview Total 10-12 IDIs	• Quality of care (safety, patient centeredness, equity, access, timeliness) • Satisfaction and adherence to intervention. • Factors contributing to use/refusal of intervention. • Suggestions for modification.
Physicians Nurses, organisation leaders and implementers	In-depth Interviews 10-12 interviews in each category (20- 24 total interviews)	• Quality of care (safety, patient centeredness, equity, access, timeliness) • System related barriers for adoption of intervention. • Barriers at patient level. • Satisfaction and suggestions for modifications.

**Table 5.** Performance indicators in TIME-HF.

Performance indicator	Description
*In-patient measures*	
Evaluation of left ventricular systolic (LVS) function	Proportion of HF patients with LVS function assessed before arrival, during hospitalisation, or is planned after discharge.
Angiotensin-converting enzyme inhibitor (ACEI), or angiotensin receptor blocker (ARB) for left ventricular systolic dysfunction (LVSD)	Proportion of HF patients with LVSD and without both ACEI and ARB contraindications who are prescribed ACEI or ARB at hospital discharge.
Anticoagulant at discharge for HF patients with atrial fibrillation (AF)	Proportion of HF patients with AF and without contraindications who are prescribed warfarin or NOACs at discharge.
Clinical handover	Proportion of HF patients discharged home with written instructions addressing all of the following: activity level, diet, discharge medications, follow-up appointment, weight monitoring, and what to do if symptoms worsen.
Adult smoking cessation advice/counselling	HF patients with a history of smoking cigarettes, who are given smoking cessation advice or counselling during hospital stay.
*Outpatient measures*	
LVS function assessment	Proportion of HF patents with documentation that LVS has been assessed.
Weight measurement	Proportion of patients with measurement of weight at each outpatient visit to assess change in volume status.
Blood pressure measurement	Measurement of patient’s blood pressure and calculation of pulse pressure at each outpatient visit.
Assessment of clinical signs and symptoms of volume overload (excess)	Assessment of clinical symptoms of volume overload at each outpatient visit (e.g., dyspnoea, orthopnoea). Signs include peripheral oedema, rales, hepatomegaly, and ascites. Proportion of patients without hypoperfusion and congestion.
Assessment of activity level	Proportion of patients with evaluation of the impact of HF on activity level at each outpatient visit.
Patient education	Percentage of patients who were provided with patient education on disease management and health behaviour changes during follow-up visits.
Beta-blocker therapy	Prescription of beta-blockers in patients with HF and LVSD. Adherence to Beta- blocker therapy at follow-up visit.
ACEI or ARB therapy for patients with HF who have LVSD	Proportion of patients with prescription of ACEI or ARB for management of outpatients with LVSD. Adherence to ACEI or ARB therapy at follow-up visit.
MRA for patients with HF	Prescription of MRA for management of outpatient with LVSD. Adherence to MRA therapy at follow-up visits.
Warfarin therapy for patients with AF	Proportion of HF patients with chronic/recurrent AF and without contraindications who are adherent to warfarin/NOACs at follow-up.
Assessment of depression	Proportion of patients with assessment for depression

**Trial sample size and power**

The mean difference in DAOH was assumed to be 20 days (586Vs 566 days with a standard deviation of 69 days) at two-year. We used the DAOH at two-year from the Trivandrum Heart Failure Registry ^7^. In the intervention group, we assumed 3% higher DAOH. A sample size of 770 HF participants per group (a total of 1540), in 11 equal clusters per arm (total 22 clusters) provides 91% power for a 2-sided 5% alpha. Assumptions; an ICC of 0.01 (design effect 2.08), coefficient of variation of cluster size of 0.85.

**Study coordination**

Sree Chitra Tirunal Institute of Medical Sciences and Technology (SCTIMST), Trivandrum, India will be the study coordinating centre and trial sponsor. There will be 22 participating centres in India including SCTIMST. Hospitals with facilities for management of HF will serve as the cluster units or participating centres. Each participating centre will be recruiting 70 patients.

**Data entry and data management**

Data will be collected by trained nurses using study questionnaire. Training will be provided for collection of clinical data and personal data from the patients. Nurses under the supervision of a principal investigator and post-doctoral fellow will do data entry on Redcap application using a tablet computer. The data will be cleaned, queries enquired and analysed by post-doctoral fellow and research fellows. The study will collect both quantitative and qualitative data. The data will be de-identified to make sure confidentiality of the data.

**Data analysis plan**

All quantitative analysis will follow guidelines of cluster randomised trials ^53^. We will employ intention to treat analysis except in the sub-group analyses. Initially, baseline characteristics will be compared by treatment group to examine the adequacy of randomisation. The primary analysis will be a complete case analysis. However, missing data on outcome variables will be reported and sensitivity analyses will be conducted after multiple imputation of missing data.

The DAOH will be calculated by subtracting days in hospital and days from death until end of study follow-up from the total follow-up time of 730 days. Empirical density curves will be created to show the distribution of DAOH over two-year follow-up period, stratified by intervention group. The median and the interquartile range of DAOH by treatment group will be provided. Poisson regression with a robust variance estimate ^54^ and an offset term to account for clustering will be employed in the analyses of DAOH per two-years of follow-up time. This procedure will yield a rate ratio and 95% CI ^54^. The rate ratio is the DAOH in the intervention arm divided by DAOH in the enhanced usual care arm. A rate ratio of >1 indicates more DAOH in the intervention arm in comparison to the usual care arm (i.e., favours the intervention).

Multilevel mixed-effects survival models will be employed for analyses of time to secondary outcomes (composite of mortality and hospitalisation). A random term identifying the location of participating sites will account for the clustering effect. We will also conduct a landmark analysis conditional upon intervention group membership at 30 days of follow-up. The between-group differences for each of the secondary outcomes (other than binary outcomes variables) will be measured using mixed-effect linear models and after accounting for clustering of observations. Standard errors will be calculated using robust estimation procedures ^55^.

Pre-specified sub-group analyses (age group, sex, region, type of facility, clinical severity) will be conducted. The significance of subgroup effects will be assessed by tests of interactions of covariates and the treatment effect.

Finally, as an exploratory analysis we will also use win ratio ^56^ to analyse the composite secondary outcome of mortality and hospital admissions. The win ratio statistic prioritises the mortality and hospitalisation endpoints through sequential comparisons. Patients in the intervention and usual care group will be converted into matched pairs based on their baseline risk profiles. The intervention patient will be labelled as a ‘winner’ or a ‘loser’ depending on who died first. If there is no death, the pairs will be labelled a ‘winner’ or ‘loser’ depending on who had a hospitalisation first. Otherwise, they are considered tied. Finally, the win ratio will be generated, which is the total number of winners divided by the total numbers of losers. A 95% confidence interval and *P*-value for the win ratio will be obtained. If matched pairs are not possible; the analyses will be conducted on unmatched pairs.

The data on costs for the intervention and control groups will be compared to assess Incremental Cost-Effectiveness Ratios (ICER), the differences in outcomes between the intervention and control groups versus differences in costs of the intervention components. ICER measures will include the cost per case of primary outcome avoided. If the primary clinical outcomes are shown to differ significantly between group, a full economic evaluation of the lifetime costs, benefits, and cost-effectiveness (in life years gained) comparing the usual care to intervention strategy will be performed. Decision models from health system and societal perspectives, a lifetime analytic horizon, and 3% discounting of future costs and outcomes will be used. QALYs will be derived from EQ5D-VAS. We will also estimate the economic rate of return of an additional rupee spent on the intervention, with the return being in the form of knock-on costs of health services saved. For this purpose, the costs of the intervention will be the direct and indirect costs for the intervention components, but excluding knock-on costs on health service use. The differences in the costs of health service use in the treatment and control participants will be used to construct an estimate of monetary savings. The ratio of these (discounted using 3%) savings and intervention costs, will be used to derive the economic rate of return over 2-years from the start of the intervention.

**Study outcomes**

Primary outcome is the days alive and out of hospital (DAOH) during the two-year follow-up period. Major secondary outcomes include; a) a composite endpoint of mortality (all-cause) or hospitalization (>24 hours) during study follow-up period, b) six minutes walking distance, c) adherence to GDMT and d) quality of life.

**Data safety and monitoring**

Data will be reviewed on real-time basis and feedback will be provided to the participating sites. Periodic monitoring of the data will be done once in six months. Source data verification of 10 percent of the data fields will be conducted. A data safety monitoring board (DSMB) ^57^ with members independent of the trial will review the trial outcome and safety data annually.

## Discussion

Heart failure is a chronic condition with a wide range of effects on the activities of daily living and the need for lifelong management. There have been considerable advancements in the treatment and management of HF in the recent past. Despite these developments HF patients still experience high treatment burden, reduced quality of life, frequent hospitalisations and death ^58^. A team-based approach involving task sharing with different cadres of health care providers may be best suited for management of a multimorbid condition like heart failure.

Guideline directed medical treatment is the main pillar of chronic management of HF with reduced EF ^59, 60^. One of the challenges in HF management is the implementation of complex treatment regimens especially for those with co-morbidities and the effective tracking of patients to monitor the disease progression. A patient who has been initiated on GDMT needs careful monitoring and close follow-up for titration of the medication. Although the benefits of GDMT in the management of HF has been documented, there exists a gap in the provisioning and the adherence of GDMT ^61^. The physician driven surveillance and management of the HF patients is often difficult due to their busy clinical schedules. At the patient level, the ability of the patients to travel to the clinic to manage their conditions is a hindrance and therefore results in delay in such measures especially in low resource settings.

Timely monitoring of the symptoms of congestion and the effective implementation of healthy behaviours into the daily lives are some other challenges in HF management ^62^. For the patients to adhere to treatment guidelines and lifestyle modification, a targeted intervention is needed. Given the effectiveness of task sharing strategy of nurses in management of cardiovascular conditions in different settings ^17, 18^, monitoring patients remotely with a specially trained nurse facilitating the management, and nurses acting as care coordinator in a team-based care model with support from physicians and other health care providers may be a viable strategy to improve HF outcomes in low resource settings.

The mHealth application facilitates real time monitoring of the warning signs and symptoms of worsening HF. The patients will be advised to update risk factors, anxiety levels, depression, warning signs and symptoms of heart failure through the mHealth application. In addition to the daily monitoring of the patients they are also advised to report a weekly survey. This can help the nurses and the doctor to make informed and timely decisions on management of the patients and prevent the need for hospitalisation due to exacerbations or worsening of the condition.

Although collaborative care has been a successful model in high-income settings for management of chronic conditions, there is still a lack of understanding on the acceptability, and feasibility of it among patients and providers in India and other LMICs ^63^. Our trial will explore the acceptability and feasibility of CCM the Indian settings. We will also investigate the overall cost-effectiveness of the intervention strategy. The effect of CCM on diseases specifically mental health conditions like depression and anxiety has established it as an effective model for care ^64, 65^. Since heart failure is a multimorbid condition and often co-exists with mental health conditions, CCM may have important role in improving the quality of life, and physical functions of the patients compared to routine care. In our trial, the CCM will be developed based on the inputs from various stakeholders like doctors, nurses, patients, and their caregivers. This will help us to design a patient centred approach in management of HF.

### Implications

The findings of TIME-HF trial will have the potential for changing the care delivery of HF and other chronic conditions in India. The knowledge generated from TIME-HF study will identify the system-level changes needed to address the limitation of the current care for HF. The collaborative care model has the potential to improve the communication and collaboration between specialists, nurses, and other stakeholders for a comprehensive care delivery for HF. The remote monitoring of the patients, early identification of the warning signs and symptoms of worsening HF, and timely management may help to prevent hospitalisation and mortality.

### Dissemination

The key-findings will be published in leading academic journals as well as it will be presented in conferences. Policy implication of the study findings will be developed, and it will be shared with various stakeholders at the state, regional and national level. 

### Study status

At the time of protocol submission, all participating sites had been identified and recruitment of patients had started. The data collection is planned to be completed by end of 2024.

## Data Availability

No data are associated with this article. Figshare: Structured Questionnaire https://doi.org/10.6084/m9.figshare.21802917.v1 ^37^ The file “Structured Questionnaire” contains the following extended data: Baseline Proforma 3 ^rd^ , 6 ^th^, 9 ^th^, 12 ^th^, 15 ^th^, 18 ^th^, 21 ^st^, 24 ^th^ month follow-up questionnaire Figshare: 7 ^th^ day Follow-up Questionnaire https://doi.org/10.6084/m9.figshare.21802815.v2 ^38^ The file “7 ^th^ day Follow-up Questionnaire” contains the following extended data 7 ^th^ day follow-up questionnaire for intervention arm only Figshare: Informed Consent (TIME-HF study) https://doi.org/10.6084/m9.figshare.22360375.v1 ^29^ The file “Informed Consent (TIME-HF study)” contains the following extended data Participant information sheet and Consent form Figshare: List of participating centres https://doi.org/10.6084/m9.figshare.22360585 ^35^ Figshare: Data Safety Monitoring Board https://doi.org/10.6084/m9.figshare.22360561 ^57^ Figshare: Timeline of TIME-HF trial https://doi.org/10.6084/m9.figshare.22360615 ^32^ Data are available under the terms of the Creative Commons Attribution 4.0 International license (CC-BY 4.0).
