# Supplementary material for: Team based collaborative care model, facilitated by mHealth enabled and trained nurses, for management of heart failure with reduced ejection fraction in India (TIME-HF): design and rationale of a parallel group, open label, multi-centric cluster randomised controlled trial
Source: Wellcome Open Res. 2023 Jul 12;8:197. Originally published 2023 May 3. [Version 2] doi: 10.12688/wellcomeopenres.19196.2 (PMC10545985; doi:10.12688/wellcomeopenres.19196.2)
Supplement: Peer review history

# Reviewer report: Reviewer response for version 2

**DOI:** 10.21956/wellcomeopenres.21784.r62627

**Published:** 2023-10-02

**Authors:** Heather Ross

**Affiliations:** 1 Edson College of Nursing and Health Innovation, Arizona State University, Tempe, Arizona, USA

Thanks to the authors for their detailed response to the previous review. The changes and acknowledged limitations are duly noted and appropriately represented.

Is the study design appropriate for the research question?

Partly

Is the rationale for, and objectives of, the study clearly described?

Yes

Are sufficient details of the methods provided to allow replication by others?

No

Are the datasets clearly presented in a useable and accessible format?

Not applicable

Reviewer Expertise:

Cardiology, implementation science.

I confirm that I have read this submission and believe that I have an appropriate level of expertise to confirm that it is of an acceptable scientific standard.

# Reviewer report: Reviewer response for version 1

**DOI:** 10.21956/wellcomeopenres.21277.r57148

**Published:** 2023-06-14

**Authors:** Devraj Jindal

**Affiliations:** 1 Centre for Chronic Disease Control (CCDC), New Delhi, Delhi, India

This study, conducted by Jeemon P *et al *between September 2021 to August 2026 focuses on the implementation of a team-based Collaborative Care Model (CCM) which would be guided by a trained nurse, for the management of Heart Failure, focusing on Days Alive and Out of the Hospital (DAOH), with the support of mHealth and to evaluate their acceptability and effectiveness in Indian setting.

****

 The study plans to use a formative qualitative research for the purpose of identifying barriers and facilitators for implementing a team-based CCM for heart failure management; a multi centric randomised controlled trial to assess the effectiveness of the CCM in improving DAOH, cost effectiveness and evaluative qualitative research to understand the scalability of the intervention model in terms of cost-effectiveness, provider satisfaction, and patient outcomes.

 The initial step is to conduct in-depth interviews with multiple stakeholders involved in the Heart Failure Management, the key findings of which would be helpful in making the intervention contextually relevant. For the CCM intervention, the study will conduct a parallel group cluster Randomised Controlled Trial with more than 1500 Heart Failure Participants from 22 units in India, each unit being randomly assigned as intervention group (CCM) and control group (standard care).There will be a rolling recruitment for 6-9 months and a structured questionnaire will be collected by the trained research nurses, at baseline and every 2 months for a period of 2 years. The CCM will be delivered in 3 phases-risk stratification, lifestyle modifications and disease management with the aid of mHealth technology (remote monitoring and decision prompts). The mHealth application facilitates real time monitoring of the warning signs and symptoms of worsening Heart Failure.

 Economic analysis will be conducted during the trial, every 6 months, with a top down approach for Health Systems perspective and bottom up approach for patient-level costs. At the end of treatment, the study will assess the perspectives of patients and nurses on the quality of care, intervention barriers, and participation benefits or disadvantages.

 The primary outcome of the study over the two-year follow-up period will be DAOH, while the major secondary outcomes include a composite endpoint of mortality or hospitalization (>24 hours), 6-minute walking distance, adherence to Guideline Directed Medical Therapy (GDMT), and quality of life.

 In summary, this trial aims to develop a Comprehensive Care Model (CCM) for Heart Failure management in India by incorporating input from various stakeholders, such as doctors, nurses, patients, and caregivers. The goal is to establish a patient-centred approach that prioritizes the individual needs and preferences of Heart Failure patients. The study findings have the potential to impact the delivery of healthcare for Heart Failure in India.

 The authors have provided important and detailed information in the body of the manuscript. The objective, study design, and methods are clearly described. However, it would be good to know more about the intervention. I have a few queries related to the intervention:

- What are the qualifications of the nurses involved? Will these nurses be specifically hired for the project, or will existing resources be utilized for intervention delivery?
- More details about the mHealth component would be useful. Is the mHealth component compliant with the Ayushman Bharat Digital Mission (ABDM)?

Overall, the research is going to be useful for providing insights and solutions for quality care delivery for heart failure in India.

Is the study design appropriate for the research question?

Yes

Is the rationale for, and objectives of, the study clearly described?

Yes

Are sufficient details of the methods provided to allow replication by others?

Yes

Are the datasets clearly presented in a useable and accessible format?

Not applicable

Reviewer Expertise:

I am a public health specialist with more than ten years of experience utilizing technology and applied research at both the health system and community levels. My extensive background includes progressively designing, implementing, and leading user-centered digital health interventions that aim to enhance health outcomes.

I confirm that I have read this submission and believe that I have an appropriate level of expertise to confirm that it is of an acceptable scientific standard.

# Response

**Published:** 2023-06-26

**Authors:** Panniyammakal Jeemon

**Affiliations:** Sree Chitra Tirunal Institute for Medical Sciences and Technology, Trivandrum, India

Thank you very much for the positive feedback on the study protocol and the trial design. As suggested, we have added more details on the mHealth application in the manuscript text along with study intervention overview section. The patient's mHealth application is made available in the Google Play Store. We are not sure whether the mHealth component is currently compliant with the Ayushman Bharat Digital Mission (ABDM). Our technical team will evaluate the requirements and they will ensure that it is compliant with ABDM in the next revision. Thank you for this valuable suggestion.

# Reviewer report: Reviewer response for version 1

**DOI:** 10.21956/wellcomeopenres.21277.r56945

**Published:** 2023-06-05

**Authors:** Heather Ross

**Affiliations:** 1 Edson College of Nursing and Health Innovation, Arizona State University, Tempe, Arizona, USA

Thank you for the opportunity to provide peer review of the study protocol prepared by Jeemon and colleagues to describe the evaluation of a team based collaborative care model for management of heart failure in India. The proposed research study seeks to employ a cluster RCT design to evaluate the effectiveness of implementing a nurse-led team-based collaborative care model for managing heart failure patients at clinics in India. This review will comment on both strengths and opportunities to strengthen the work with particular attention to the rationale, study design, and methods described in the study protocol. Several typographical errors including omitted words and errors in subject-verb agreement were noted throughout the manuscript. This review will not enumerate those, as the authors can identify and fix the issues that were likely simply an oversight.

Rationale for the Study

 The authors rightly identify physician workforce challenges to address the complex work of managing patients with heart failure in the outpatient setting, along with strong evidence supporting the efficacy of nurse-led interventions to prevent re-hospitalization compared to usual care. The attention to evidence related to low and middle-income countries, and evidence from India specifically, is noted and appreciated. I note that the literature review mentions three meta-analyses, but only two are cited.

 The identification of the collaborative care model (CCM) based on Wagner’s Chronic Care Model is an appropriate theoretical model to guide the work. I note that the authors’ phrasing suggests that the team-ness of their CCM approach is novel. However, team approaches are inherent to CCMs, and indeed Wagner’s Chronic Care Model specifies a team as the healthcare delivery mechanism. With that in mind, the authors may wish to revisit the introduction section to expand the literature review to include evidence pertaining to team-based care in addition to the literature review on nurse-led care.

 In addition to the Chronic Care Model as a theoretical model, I note that the proposed study seeks to implement an intervention with established effectiveness evidence, that is, the nurse-led team-based heart failure outpatient management approach. As such, the authors may wish to consider an implementation science approach (PRISM/RE-AIM) as the most appropriate framework to guide the study design.

 The paragraph describing study aims would benefit from some reorganization. The sentences expanding on DAOH following aim 2 are out of place in a list of aims and may be better situated in the methods section discussion of measures. In addition, the sub-letters for aim three are not clearly related to the stated overarching aim focused on scalability. As such, the research team may wish to reconsider the notion of scalability as the overarching aim. Here, the recognition of an implementation science framework would be appropriate as it explicitly attends to issues of reach, effectiveness, adoption, implementation, and maintenance of the intervention in the target setting.

Study Design

 The mixed-methods approach using qualitative interview including multiple stakeholder groups (Aim 1) to inform the design of a cluster RCT (Aim 2) is appropriate. The cost-effectiveness and evaluative qualitative research (Aim 3) following the RCT may be appropriate. However, the evaluation methods for Aim 3 may benefit from additional clarity as will be discussed in the following section.

 The use of the SPIRIT checklist is appropriate. However, there is no clear implementation study framework noted. Again, the PRISM/RE-AIM approach would offer rigor. In addition, the protocol mentions a SPIRIT flowchart that appears to be Table 3, though it is not cited in the text in the Methods-Study Design Approach section where it is first mentioned.

 Considering the RCT (Aim 2), the study design would benefit from a description of the 22 units that will compose the RCT sites. Are these units in distinct locations or catchment areas where patients are unlikely to overlap between sites? Do the units employ teams unique members, or is there overlap between personnel that might call for mitigation to prevent cross-contamination between sites? Do the site teams have similar compositions in terms of clinical roles and functions or are they highly heterogeneous? In the case of heterogeneity, how will differences between sites be accounted and accommodated for both the intervention and for the usual care comparator? Regarding usual care, the text would benefit from a description of the present standard of care along with usual care practices at each site in order to determine that the randomized groups are not substantively different at baseline. To this point, if the control arm design follow-up plan diverges from usual care in terms of the frequency of clinical follow-up described in the study design, the divergence from usual care should be noted.

 Noting the patient eligibility criterion of reduced ejection fraction, it would be appropriate to clarify that the heart failure intervention is specific to heart failure with reduced ejection fraction (HFrEF) and does not encompass care of all patients with heart failure including heart failure with preserved ejection fraction (HFpEF). This clarification would be appropriate throughout the protocol including in the title, as the present depiction of broad heart failure inclusion may be misleading.

 Table 3 provides a helpful tool to organize the extensive data collection scheme planned for the study. It is notable that key measures relevant to quality of life assessment including PHQ-9, KCCQ 12, GAD-7, CTM-3, and B-prepared scale are measured only at baseline. These should be measured again at least at the conclusion of the study in order to properly assess the study’s impact on these measures. Similarly, the decision to omit some measures at baseline (e.g. medication adherence scale, specific activity questionnaire, weight management) raises questions about the study’s validity for assessing impact on measures that are not assessed at baseline.

Study Methods

 The protocol would benefit from additional methodological description of the qualitative interview approach for Aim 1. For example, is the planned sample size of 10-12 interviews for each stakeholder category determined by a grounded theory approach using a semi-structured questionnaire with ongoing analysis until thematic saturation is reached? Is attention paid to key informant characteristics such as geography, socioeconomic status, or other demographic characteristics when composing the sample?

 Regarding study measures, the extensive use of validated and reliable tools for Aim 2 is commendable and Table 2 is helpful and well organized. There are some listed items in the table that lack clarity, however. With regard to self-management measures, it is not clear how weight management, diet management, family support, and patient care in depression will be measured. Regarding quality of life, 6-minute walk test is not an appropriate direct measure and should be listed only under walking ability. Regarding functional capacity, more detail is needed about the specific activity questionnaire. Regarding disease severity, detail is needed about the responsiveness to change in heart failure symptoms. Regarding patient satisfaction, more detail is needed about patient satisfaction in quality of care.

 Regarding health care expenditure data, significantly more detail is needed about how data will be collected. Will a structured questionnaire be used, and if so will that questionnaire have established validity and reliability? Given the placement of the sentence about health care expenditure data in a paragraph that begins with multimorbidity treatment burden and continues with blood pressure, should it be assumed that this reference to expenditure data will be at the level of the individual patient, and not at the institutional level?

 Regarding the description of the study intervention, how will the clinical teams be trained in the intervention? How will fidelity of the intervention be assessed? What is entailed in the mHealth assisted disease management program? Is it a commercially available system? Is it already in use in the target systems? What types of biometrics will it monitor? How does it signal healthcare providers? How does the patient interact with it? Significantly more detail is needed on all of these points.

 Regarding Aim 3, the mention of questionnaires that have previously been used in South Asian contexts to assess indirect costs occurred by patients is appreciated and merits more discussion. Do these questionnaires have established validity and reliability? A citation for the questionnaire is needed. In addition, the description of methods for Aim 3 mentions that patients and nurses will be queried about quality of care and other items. Table 4 indicates that investigators will use “in-depth interview.” As with the interviews mentioned for Aim 1, it would be helpful to share more precise information about the qualitative interview methods. Moreover, there may be suitable questionnaires with established validity and reliability to assess some of the items (e.g. quality of care) that may be appropriate to consider. Regarding the additional close-out questionnaire mentioned, more detail is needed about established validity and reliability, along with a citation.

 Regarding data analysis, the analytical plan for qualitative data is missing entirely and must be added for both the qualitative interviews planned for Aim 1 and Aim 3.

Is the study design appropriate for the research question?

Partly

Is the rationale for, and objectives of, the study clearly described?

Yes

Are sufficient details of the methods provided to allow replication by others?

No

Are the datasets clearly presented in a useable and accessible format?

Not applicable

Reviewer Expertise:

Cardiology, implementation science.

I confirm that I have read this submission and believe that I have an appropriate level of expertise to confirm that it is of an acceptable scientific standard, however I have significant reservations, as outlined above.

# Response

**Published:** 2023-06-26

**Authors:** Panniyammakal Jeemon

**Affiliations:** Sree Chitra Tirunal Institute for Medical Sciences and Technology, Trivandrum, India

Thank you for the detailed review and valuable comments on the protocol paper. We have made appropriate corrections in the protocol as suggested. The changes made in the protocol are summarised below.

 Rationale for the study:

 We have provided the details of one of the missing meta-analyses and cited the article appropriately in the background/introduction section. We have also removed the expansion of DAOH and inserted along with methods section.

 We agree that team-based care is inherent to the CCM. However, team-based care with structural changes in the health care delivery mechanism in the LMIC context for management of heart failure is new. We have reorganised the study background section with more focus on the CCM. We have also explained the context in India with additional details in the methodology section. We thank the reviewer for their suggestion to consider RE-AIM framework. Although CCM is reasonably well established in high-income settings, this model with the support of mHealth is not tested in LMIC settings especially among HF patients. We have highlighted this point and focused more on efficacy of our approach than effectiveness. We have used evidence from multiple studies to develop an intervention model that is context specific for India. Further, we utilised the MRC framework for development, implementation, and evaluation of complex public health interventions. Our intervention strategy satisfies the definition of complex public health intervention. We have however, incorporated components of RE-AIM (especially adoption, and maintenance) in the process evaluation. We have added these details in the study design approaches as part of the methods section. We have limitations in further modifying the methodology as it is a funded and ongoing study. 

 Study design:

 Response: In the study design section, we have made the necessary correction as suggested. We appreciate the comments on the inclusion criteria. We are recruiting only heart failure patients with reduced ejection fraction. We have made the corrections in the title and in the protocol text to reflect that the trial is specific to heart failure patients with reduced ejection fraction.

 For Aim 1, the number of interviews in the qualitative component will be decided based on the thematic saturation for each stakeholder. We have included stakeholders both from facilities with and without dedicated HF centres. The necessary changes on the rationale for the number of interviews for each stakeholder and the selection of type of stakeholders have been added in the study design section.

 For aim 2, the selected units are independent units with dedicated staff employed at each site. Further, the mHealth application would be only available for the selected participant in the study. Therefore, the chance of contamination is minimum. However, patients may get discharged from one hospital and go to another hospital for follow-up care. We would not be able to prevent such cross-over if that happens in the study. However, it will be documented and considered in separate per-protocol analysis if necessary. We have incorporated the description of the units, measures to mitigate the cross-contaminations and handling of heterogeneity across the units in the study design section.

 Timing of administration of tools:

 We apologise for the oversight, and we have made appropriate changes in table 3 and the manuscript text. The PHQ-9, GAD-7, KCCQ, CTM-3 and B-prepared scale will be repeated at 12-month and 24-month. Some of the measures are not collected at baseline since the baseline data are collected at discharge after an admission. For example, we may not be able to ask question regarding adherence at the time of discharge. Similarly, some tools are specific for the hospital admission and discharge procedures adopted. It will be administered only during the time of discharge from the hospital. We have separate structured questionnaires for assessing weight management, diet management, family support and patient care in depression. Similarly, specific activity questionnaire, responsiveness to change in heart failure symptoms and patient satisfaction in quality of care are also incorporated as structured questionnaires. We have provided all these tools for data collection in the online supplementary files.  

 Thank you for identifying the missing citations in Table 2. We have incorporated those missing citations and added them in our reference listing. It was an oversight to include the six-minute walking test along with quality of life. We have made corrections appropriately. The health care expenditure data will be captured using a treatment expenditure questionnaire. It is provided in the online supplement. We have also added the appropriate reference for the questionnaire and cited it in the manuscript text. This tool has been used in the south Asian context for economic evaluation. As noted in the comments, the expenditure data will be measured at the individual patient level  and not at the institution level. We have added the details of the mHealth application in the study intervention overview section. The mHealth application is not commercially available. The patient application can be downloaded from Play store but the registration to the application can only be done with the help of the nurse in the project through another application – nurse app. Nurse app is not available in Play store, and it is available to the concerned nurse in the project via invitation. This is to prevent contamination and potential use of the application by other providers and patients.

 We thank the reviewer once again for their insightful comments.   
